# *In Vitro* Antimicrobial Activities of Tigecycline, Eravacycline, Omadacycline, and Sarecycline against Rapidly Growing Mycobacteria

**DOI:** 10.1128/spectrum.03238-22

**Published:** 2022-12-08

**Authors:** Tingting Zhang, Jian Du, Lingling Dong, Fen Wang, Liping Zhao, Junnan Jia, Congli Wang, Mengli Cheng, Xia Yu, Hairong Huang

**Affiliations:** a National Clinical Laboratory on Tuberculosis, Beijing Key Laboratory for Drug-Resistant Tuberculosis Research, Beijing Chest Hospital, Capital Medical University, Beijing Tuberculosis and Thoracic Tumor Institute, Beijing, China; Johns Hopkins University School of Medicine

**Keywords:** antimicrobial activity, eravacycline, omadacycline, sarecycline, tigecycline, rapidly growing mycobacteria

## Abstract

Infections caused by rapidly growing mycobacteria (RGM) have increased globally. Chemotherapy against these infections is challenging due to the minimal antimicrobial choices available. The main aim of this study was to evaluate the *in vitro* susceptibilities of four tetracyclines against different RGM species. The MICs of eravacycline (ERC), omadacycline (OMC), sarecycline (SAC), and tigecycline (TGC) against the reference strains of 27 RGM species and 121 RGM clinical isolates were determined by microtiter plate assay. The minimum bactericidal concentrations (MBCs) and cytotoxicities of these antibiotics were also tested. Except for SAC, the other three tetracyclines had MICs of ≤0.5 μg/mL against all 27 RGM reference strains. ERC generally presented the lowest MICs, with MIC_90_s against the clinical isolates of Mycobacterium abscessus subsp. *abscessus*, Mycobacterium abscessus subsp*. massiliense*, and Mycobacterium fortuitum of 0.25 μg/mL, 0.25 μg/mL, and 0.06 μg/mL, respectively. TGC and OMC also showed equivalent *in vitro* inhibitory activities against the isolates, while the TGC MIC_90_s for M. abscessus subsp. *abscessus*, M. abscessus subsp*. massiliense*, and M. fortuitum were lower than or equal to the OMC MIC_90_s (1, 1, and 0.25 μg/mL versus 1, 2, and 2 μg/mL). In addition, the MIC_50_s of three of the antibiotics for each species were always 2-fold lower than the corresponding MIC_90_s. MBC and cytotoxicity assays indicated that all four tetracycline antibiotics tested were bacteriostatic agents with low toxicity to the THP-1 cell line. Tetracycline antibiotics are efficacious in RGM infection treatment, with omadacycline showing the best promise for clinical application due to its potent antimicrobial activity, safety, and convenient administration route.

**IMPORTANCE** The global rise in antibiotic-resistant nontuberculous mycobacteria has prompted the urgent need for new antimicrobials, especially oral antibiotics. Currently, adverse effects have limited the use of tetracycline-class antibiotics, particularly tigecycline (TGC), in the treatment of rapidly growing mycobacteria (RGM). However, several new tetracycline-class antibiotics might overcome the limitations of TGC. We assessed the *in vitro* antibiotic susceptibilities of four tetracyclines (eravacycline, omadacycline, sarecycline, and tigecycline) against reference RGM strains and clinical isolates of different RGM species. We showed that three of these antibiotics (tigecycline, eravacycline, and omadacycline) might be efficacious in M. abscessus subsp. *abscessus*, M. abscessus subsp. *massiliense*, and M. fortuitum treatment. Furthermore, omadacycline was more promising for clinical application for M. abscessus infections as an oral drug, whereas sarecycline, which had the best safety parameters, should be considered a potential antibiotic for M. abscessus infections caused by susceptible strains. Our work underscores the possible clinical applications of tetracycline-class antibiotics in the treatment of RGM infections.

## INTRODUCTION

Nontuberculous mycobacteria (NTM) are recognized as important opportunistic pathogens of humans that can cause pulmonary infection, lymphadenitis, skin abscesses, disseminated infection, and systematic infection. In recent years, an evident increase in the prevalence of NTM infection has been reported globally, and it has even surpassed tuberculosis (TB) in given countries (1–5). In general, NTM can be categorized as rapidly growing mycobacteria (RGM) or slowly growing mycobacteria (SGM) based on the speed of growth. Mycobacterium abscessus complex and Mycobacterium fortuitum are among the most frequently isolated and pathogenic RGM (2, 3, 5). M. abscessus complex often causes severe, chronic pulmonary infections in susceptible hosts, and soft tissue infections caused by it have also been reported frequently (6, 7). The treatment success rate with M. abscessus pulmonary disease (MAB-PD) is an unsatisfactory 45.6% (127/221), because of the high frequency of mutational and acquired resistance to the macrolides that are the basis of chemotherapy (8). The M. abscessus complex is the most frequently encountered RGM group in many countries (7, 9, 10). The M. abscessus complex consists of M. abscessus subsp*. abscessus*, M. abscessus subsp*. massiliense*, and M. abscessus subsp*. bolletii.* Diverse susceptibilities to certain antibiotics within the complex have been reported. For example, the *erm* gene, encoding the ribosomal methylase, leads to inducible resistance to macrolides. M. abscessus subsp. *abscessus* and subsp. *bolletii* confer inducible resistance to macrolides by expressing a functional *erm* gene, whereas most isolates of M. abscessus subsp*. massiliense* show intrinsic susceptibility to clarithromycin with a nonfunctional *erm* gene (8, 11, 12). M. fortuitum can cause soft tissue infection during trauma and surgery, while associated lung diseases are rare (13). Currently, only a few antibiotics, with limited efficacies, are available for treating RGM infections, which means there is urgent need of identifying new or repurposed antimicrobials against RGMs, especially oral antibiotics.

Tetracycline-class antibiotics, particularly tigecycline (TGC), have demonstrated strong *in vitro* activity against RGM and have already been recommended to be used in the treatment of M. abscessus complex disease according to the current guidelines (7, 14, 15). A retrospective case series showed favorable responses to TGC-containing regimens in 16 of 26 patients (62%) with M. abscessus pulmonary disease (16). However, administration of TGC via intravenous infusion (i.v.) decreased patient compliance, especially due to its long course. Furthermore, the adverse effects of TGC are another important concern. It was reported that 94% of the patients who underwent TGC treatment exhibited side effects, most frequently gastrointestinal (GI) upset (16, 17). As a result, about 50% of the M. abscessus disease patients discontinued TGC-containing regimens (18).

Recently, several new tetracycline-class antibiotics were developed with the hope of overcoming the limitations of TGC. Eravacycline (ERC) is a fully synthetic fluorocycline that binds the 30S ribosomal subunit of bacteria and was approved by the U.S. Food and Drug Administration (FDA) in 2018 (19). ERC is administered intravenously to treat complicated intra-abdominal infections caused by antibiotic-resistant bacteria (20). Omadacycline (OMC) was approved by the FDA in 2018 for the treatment of community-acquired bacterial pneumonia (CABP) and acute bacterial skin and skin structure infections (ABSSI). It can be administered once daily intravenously or by oral administration (p.o.) (21). Sarecycline (SAC), an oral drug, was the first narrow-spectrum tetracycline-class antibiotic developed for acne treatment (22). Previous studies showed that SAC had fewer gastrointestinal side effects than the other tetracycline antibiotics, mainly because it is less active against the normal gut microorganisms (23–25).

In the present study, we compared the *in vitro* activities of these novel tetracycline-class antibiotics (ERC, OMC, and SAC, versus TGC), against the representative RGM to provide an additional antimicrobial section for improved treatment of RGM infections.

## RESULTS

### MICs of TGC, ERC, OMC, and SAC against 27 RGM reference strains.

The MIC results for the four tested tetracyclines are summarized in Table 1. Overall, the MICs of TGC, ERC, and OMC against the 27 RGM reference strains were all below 0.5 μg/mL, while ERC manifested the best inhibitory activities, with the majority of its MICs being ≤0.0625 μg/mL. Compared with the other tetracyclines, SAC showed weaker activities against the RGM strains, although 66.7% (18/27) of the tested reference strains had SAC MICs of ≤1 μg/mL.

**TABLE 1:** MICs of tigecycline, eravacycline, omadacycline, and sarecycline against the reference strains of 27 RGM species^*a*^

Strain	MIC (μg/mL) of:			
	Tigecycline	Eravacycline	Omadacycline	Sarecycline
Mycobacterium abscessus ATCC 19977	0.25	0.0625	0.25	>8
Mycobacterium aurum ATCC 23366	0.0313	<0.0078	0.0625	0.0313
Mycobacterium austroafricanum ATCC 33464	0.0156	<0.0078	0.0313	0.0156
Mycobacterium chelonae ATCC 14472	0.0625	0.0156	0.5	8
Mycobacterium chitae ATCC 19627	0.0156	<0.0078	0.0625	0.0625
Mycobacterium chubuense ATCC 27278	0.25	<0.0078	0.25	0.125
Mycobacterium cosmeticum DSM 44829	0.25	<0.0078	0.25	0.125
Mycobacterium diernhoferi ATCC 19340	<0.0078	<0.0078	0.125	0.25
Mycobacterium duvalii ATCC 43910	<0.0078	<0.0078	<0.0078	0.0156
Mycobacterium flavescens ATCC 14474	0.125	<0.0078	0.25	0.125
Mycobacterium fortuitum ATCC 6841	0.0156	<0.0078	0.5	2
Mycobacterium goodii ATCC BAA-955	0.0625	<0.0078	0.25	0.5
Mycobacterium mucogenicum DSM 44124	0.0313	0.0313	0.5	0.5
Mycobacterium neoaurum ATCC 25795	0.125	<0.0078	0.25	0.25
Mycobacterium parafortuitum ATCC 19686	0.0156	<0.0078	0.0625	0.0625
Mycobacterium peregrinum DSM 43271	0.0625	0.0156	0.125	>8
Mycobacterium phlei ATCC 11758	0.0625	<0.0078	0.125	0.125
Mycobacterium porcinum ATCC 33776	0.0625	<0.0078	0.125	>8
Mycobacterium pulveris ATCC 35154	0.125	<0.0078	0.125	0.125
Mycobacterium rhodesiae ATCC 27024	0.5	0.0313	0.5	1
Mycobacterium senegalense ATCC 35796	0.0156	<0.0078	0.25	0.5
Mycobacterium septicum ATCC 700731	0.0625	<0.0078	0.125	8
Mycobacterium smegmatis ATCC 19420	0.125	<0.0078	0.25	0.125
Mycobacterium thermoresistibile ATCC 19527	0.0156	<0.0078	0.25	1
Mycobacterium tokaiense ATCC 27282	0.5	0.0625	0.25	>8
Mycobacterium triviale ATCC 23292	0.0156	<0.0078	0.0625	0.0625
Mycobacterium vaccae ATCC 15483	<0.0078	<0.0078	0.0078	<0.0078

a RGM, rapidly growing mycobacteria.

### MIC distributions of the four tetracycline antibiotics against clinical isolates of M. abscessus subsp. *abscessus*, M. abscessus subsp. *massiliense*, and Mycobacterium chelonae.

**FIG 1:**
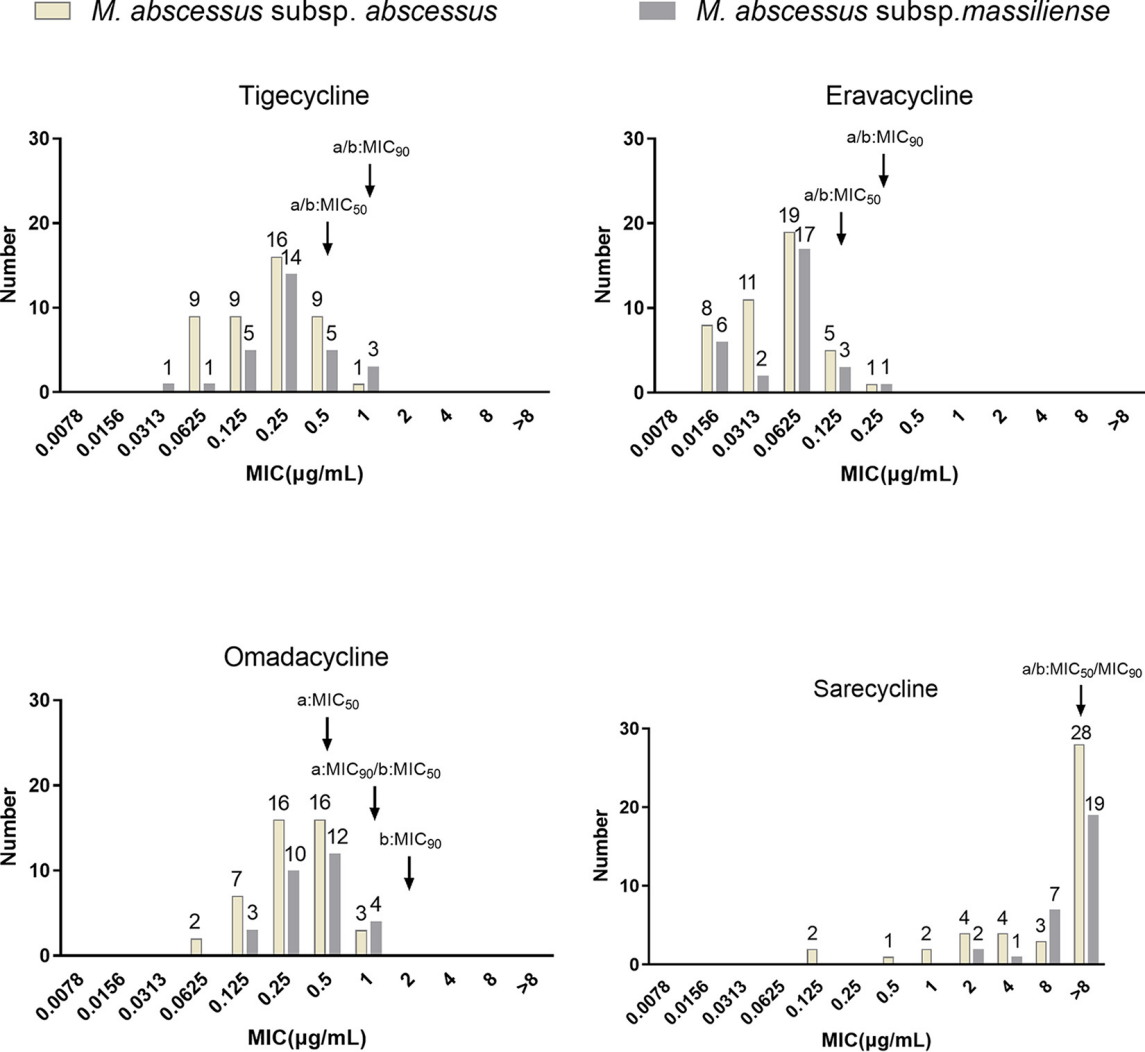
The MIC distributions of tigecycline, eravacycline, omadacycline, and sarecycline against 44 M. abscessus subsp. *abscessus* isolates and 29 M. abscessus subsp. *massiliense* isolates. The *y* axis shows the number of strains with each MIC value, with specific numbers shown above the bars. a, MIC_50_ and MIC_90_ values against M. abscessus subsp. *abscessus* isolates; b, MIC_50_ and MIC_90_ values against M. abscessus subsp. *massiliense* isolates. The software of Clinical and Laboratory Standards Institute ecoffinder-xl-2010-v21-web-version was used calculate the MIC_50_ and MIC_90_.

ERC showed the best *in vitro* activity M. abscessus subsp. *abscessus* and subsp. *massiliense*, with a MIC_90_ of 0.25 μg/mL for both (Fig. 1). Overall, M. abscessus subsp. *massiliense* seemed less susceptible to OMC, as the OMC MIC_50_ and MIC_90_ values were 2-fold higher against M. abscessus subsp. *massiliense* than against subsp. *abscessus.* Only 5 of 44 M. abscessus subsp. *abscessus* isolates and none of 29 subsp. *massiliense* isolates had SAC MICs of ≤1 μg/mL. In addition, the four tetracyclines’ MIC outcomes for the five Mycobacterium chelonae isolates presented a trend similar to that of these four antimicrobials for the M. abscessus subsp. *massiliense* isolates tested. However, M. chelonae was more susceptible to these antibiotics than M. abscessus subsp. *massiliense*, while the four antimicrobials’ MICs for the former were all less than or equal to the MIC_50_s of the latter (Table 2). The MICs of tigecycline, eravacycline, omadacycline, and sarecycline against the 44 M. abscessus subsp. *abscessus* isolates and 29 M. abscessus subsp. *massiliense* isolates are shown in Table S1 in the supplemental material.

**TABLE 2:** MICs of tigecycline, eravacycline, omadacycline, and sarecycline against five clinical isolates of Mycobacterium chelonae

Strain	MIC (μg/mL) of:			
	Tigecycline	Eravacycline	Omadacycline	Sarecycline
585	0.125	0.0313	0.25	>8
677	0.25	0.0313	0.5	1
752	0.5	0.125	0.5	>8
1392	0.5	0.0625	1	8
1593	0.25	0.0625	0.25	8

### The MIC distribution of the four tetracycline antibiotics against the clinical isolates of M. fortuitum.

**FIG 2:**
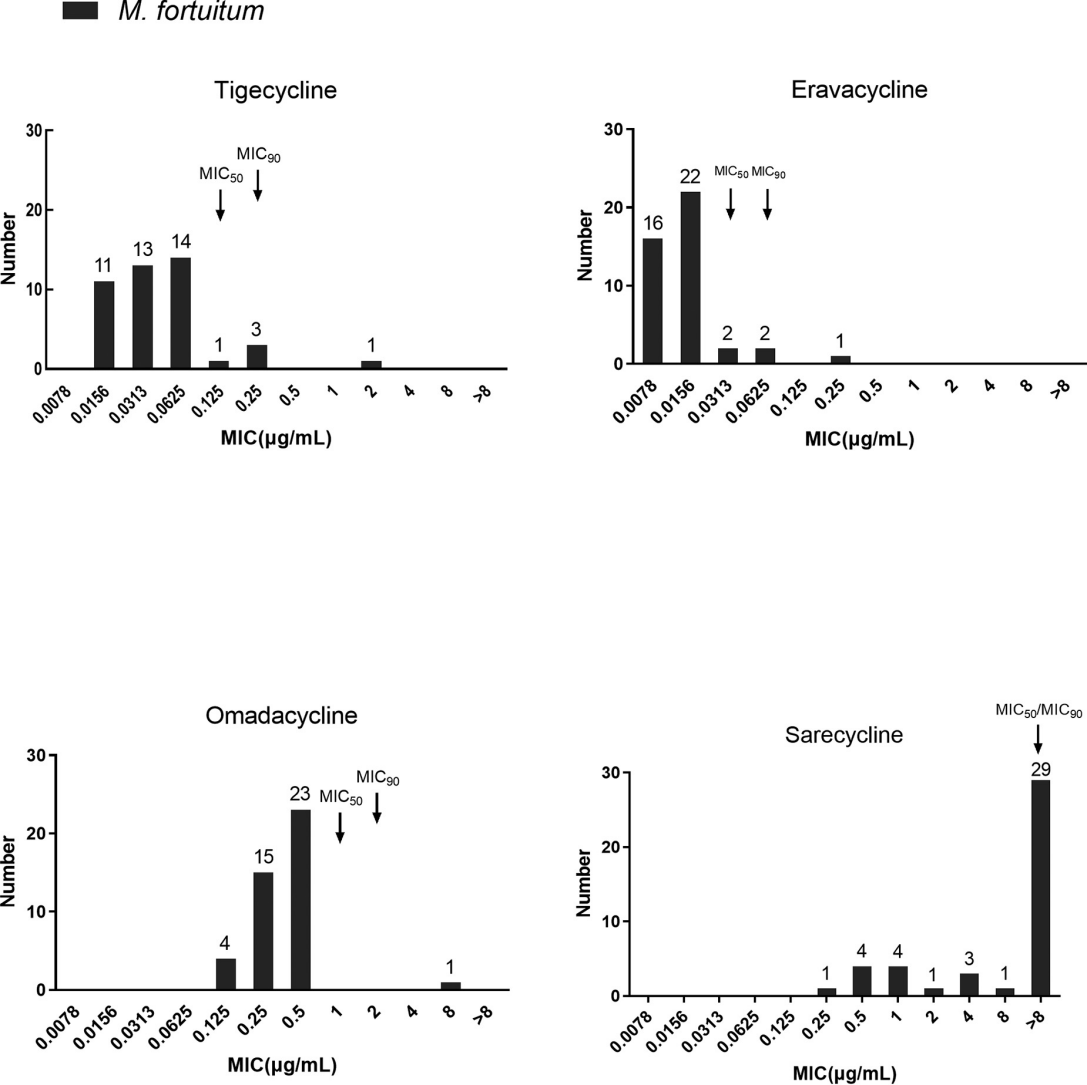
The MIC distributions of tigecycline, eravacycline, omadacycline, and sarecycline against 43 M. fortuitum isolates. The *y* axis shows the number of strains with each MIC value, with the specific numbers shown above the bars. The software of Clinical and Laboratory Standards Institute ecoffinder-xl-2010-v21-web-version was used calculate the MIC_50_ and MIC_90_.

Compared to those of M. abscessus subsp. *abscessus* and subsp. *massiliense*, M. fortuitum had 4-fold lower MICs of two of the tested tetracyclines, but not OMC and SAC (Fig. 2). ERC presented the best inhibitory activity, with a MIC_50_ and MIC_90_ of 0.0313 μg/mL and 0.0625 μg/mL, respectively, while the MIC_50_ and MIC_90_ of TGC were 0.125 μg/mL and 0.25 μg/mL respectively. Additionally, the MIC_50_ and MIC_90_ of OMC were 1 μg/mL and 2 μg/mL, respectively. However, SAC had a MIC_50_ and MIC_90_ that were both above 8 μg/mL. The MICs of OMC against M. abscessus subsp*. massiliense* (MIC_50_ of 1 μg/mL and MIC_90_ of 2 μg/mL) were generally 2-fold higher than those against M. abscessus subsp*. abscessus* (MIC_50_ of 0.5 μg/mL and MIC_90_ of 1 μg/mL). As the strain numbers for both subspecies were small, this variance needs to be validated in larger studies. The MICs of tigecycline, eravacycline, omadacycline, and sarecycline against the 43 M. fortuitum isolates are shown in Table S1.

### The MBCs of the four tetracycline antibiotics against M. abscessus subsp*. abscessus*, M. abscessus subsp. *massiliense*, and M. fortuitum.

**FIG 3:**
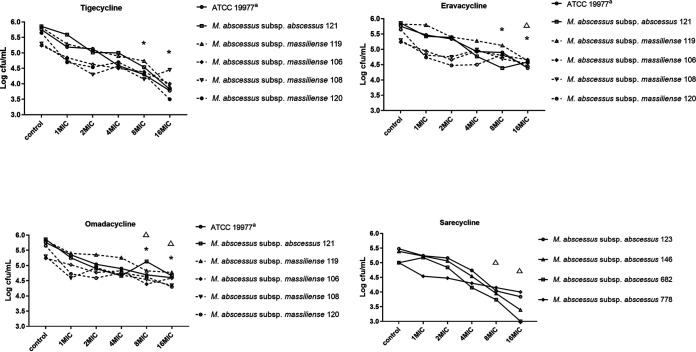
The MBCs of tigecycline, eravacycline, omadacycline, and sarecycline against the 6 M. abscessus subsp. *abscessus* and 4 subsp. *massiliense* strains identified in the keys. a, ATCC 19977 is the reference strain of M. abscessus subsp. *abscessus*. *, when comparing the Log_10_ CFU values of M. abscessus subsp. *abscessus* and subsp. *massiliense* after treatment with the tetracyclines, the values for the TGC treatment group declined significantly more than the values for the ERC and OMC treatment groups at 8× MIC and 16× MIC (two-independent-sample *t* test, *P* < 0.05); △, the Log_10_ CFU values for the SAC treatment group decreased significantly more than the values for the OMC treatment group at 8× MIC (two-independent-sample *t* test, *P* < 0.05) and significantly more than the values for the ERC and OMC treatment groups at 16× MIC (two-independent-sample *t* test, *P* < 0.05).

**FIG 4:**
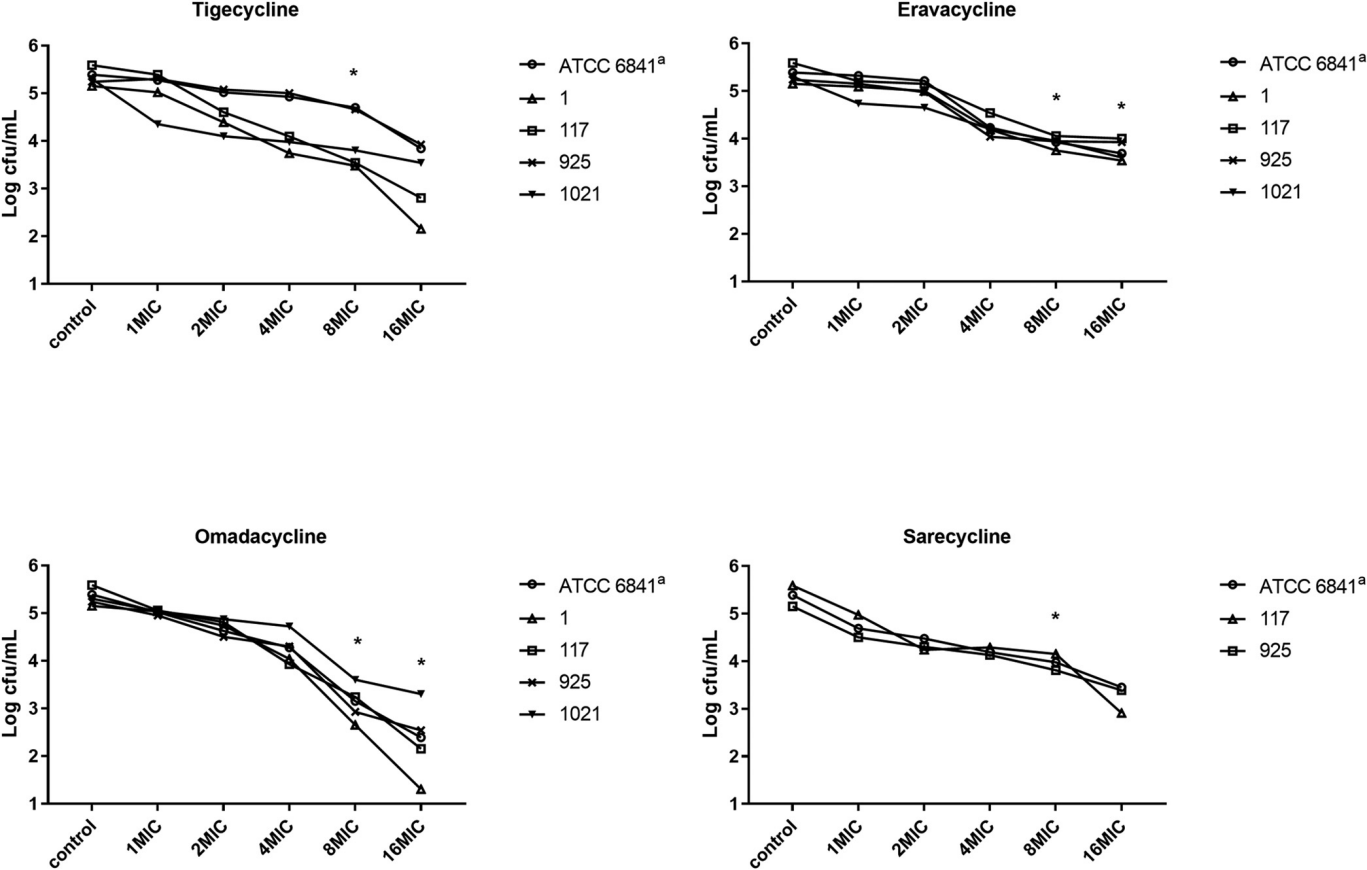
The MBCs of tigecycline, eravacycline, omadacycline, and sarecycline against the 5 M. fortuitum strains identified in the keys. a, ATCC 6841 is the reference strain of M. fortuitum. *, when comparing the Log_10_ CFU values of M. fortuitum strains after treatment with the tetracyclines, the values for the OMC treatment group decreased significantly more than the values for the TGC, ERC, and SAC treatment groups at 8× MIC (two-independent-sample *t* test, *P* < 0.05). The values for the OMC treatment group also declined significantly more than the values for the ERC treatment group at 16× MIC (two-independent-sample *t* test, *P* < 0.05).

For M. abscessus subsp. *abscessus* and subsp. *massiliense*, the MBC/MIC ratios of the four tetracycline antibiotics against the reference strain and 9 clinical isolates were all ≥16. Therefore, all four of these tetracyclines should be regarded as bacteriostatic, i.e., none of them can reduce the initial bacterial load by 99.9% CFU at 4× the MIC (Fig. 3 and Table S2). For the M. fortuitum isolates, the MBC/MIC ratios of these four tetracyclines were all ≥8 against the reference strain and 4 clinical isolates (Fig. 4 and Table S3). Therefore, all of these antimicrobials were considered bacteriostatic agents against M. fortuitum.

### *E*_max_ and EC_50_ values.

The maximum effect (*E*_max_) values of three of these tetracyclines were calculated as 47.25% (TGC), 24.27% (ERC), and 25.48% (OMC) for M. abscessus subsp. *abscessus* ATCC 19977, and the corresponding 50% effective concentration (EC_50_) values were 2.034 μg/mL, 0.253 μg/mL, and 0.441 μg/mL, respectively. In addition, the *E*_max_ values against M. fortuitum ATCC 6841 for ERC and OMC were 29.73% and 64.73%, respectively, while those for TGC were not available. The EC_50_ values for TGC, ERC, and OMC against M. fortuitum ATCC 6841 were 169.4 μg/mL, 0.026 μg/mL, and 3.353 μg/mL, respectively. Due to the high MICs of SAC against M. fortuitum ATCC 6841 and M. abscessus subsp. *abscessus* ATCC 19977 (2 μg/mL and over 8 μg/mL), its *E*_max_ and EC_50_ values could not be calculated by the current method.

### Cytotoxicity assay.

**FIG 5:**
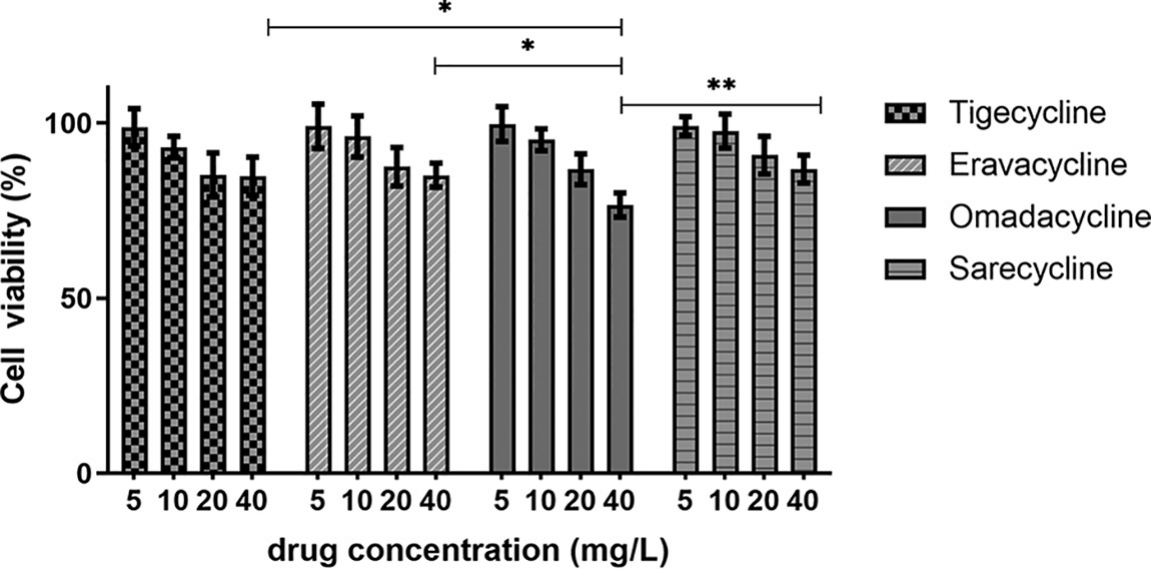
Cytotoxicity assays of tigecycline, eravacycline, omadacycline, and sarecycline in the differentiated THP-1 cell lines. All data are shown as the mean values ± standard deviations (*n* = 4). At the concentration of 40 mg/L, the cell viability for OMC was significantly lower than TGC, ERC, and SAC (two-independent-sample *t* test, *P* < 0.05). *, *P* < 0.05; **, *P* < 0.01.

According to the cell counting kit-8 (CCK-8) assay, the survival rates of THP-1 cell lines at 24 h were over 95% for ERC, OMC, and SAC at concentrations up to 10 mg/L, whereas TGC-treated cells had a lower survival rate (93.1%). At a concentration of 20 mg/L, cells treated with SAC presented the highest survival rate of 90.8%, followed by cells treated with ERC (87.5%), OMC (86.8%), and TGC (85.2%). However, at 40 mg/L, cells treated with OMC showed the lowest survival rate (76.6%). The 50% inhibitory concentration (IC_50_) values of these tetracyclines were 10.64 (TGC), 13.73 (ERC), 25.74 (OMC), and 16.83 (SAC) mg/L. In addition, at a concentration of 40 mg/L, there was a statistically significant difference in the cell viability for OMC, which was lower than the cell viabilities for TGC, ERC, and SAC (*P* < 0.05). These results are shown in Fig. 5.

## DISCUSSION

The treatment of infection caused by RGM is often long lasting, as therapy of RGM is continued for at least 12 months after sputum conversion (7). Besides this, the outcomes of RGM infection are usually unsuccessful, with low curative rates, where the sustained sputum culture conversion (SSCC) rates of the patients are 77/233 (34%) and 117/141 (54%) with M. abscessus subsp. *abscessus* and subsp. *massiliense*, respectively (26). New or repurposed antibiotics with good efficacy and safety are the major route to solving this dilemma. Several novel tetracycline-class antibiotics have been applied to treat infections in recent years, while either *in vitro* or *in vivo* activity data for these tetracyclines against different RGM species remain scarce (27–29). Limited studies have evaluated the *in vitro* activities of ERC and OMC against a few clinical isolates of M. abscessus complex (30, 31), whereas no such evaluation has been performed for SAC. To the best of our knowledge, this is the first study to compare the antibacterial activities of the four tetracyclines against RGM in parallel.

The recruited reference strains and clinical isolates of different RGM species manifested good susceptibilities to three of the tetracycline antibiotics, tigecycline, eravacycline, and omadacycline, with lower MICs (≤0.5 μg/mL). In addition, our MBC assay classified them as bacteriostatic agents against both M. abscessus subsp. *abscessus* and M. fortuitum. ERC demonstrated the most potent *in vitro* activities among the four tested tetracyclines against all the recruited RGM strains, with MIC_90_s against M. abscessus subsp. *abscessus*, M. abscessus subsp. *massiliense*, and M. fortuitum of 0.25 μg/mL, 0.25 μg/mL, and 0.0625 μg/mL, respectively. This *in vitro* activity of ERC was equivalent to or even better than that of clarithromycin, which is the core antimicrobial in the M. abscessus complex treatment regimen, with MICs ranging between 0.0625 and 2 μg/mL for susceptible strains (32). Pharmacokinetic (PK) studies have demonstrated that the maximum concentration (*C*_max_) values of ERC following intravenous administration at doses ranging from 0.5 to 1.5 mg/kg were in the range of 1.8 μg/mL to 3.4 μg/mL (33, 34). The *C*_max_ values plus the very low MICs obtained in this study imply efficacy of ERC in the treatment of RGM infections. However, ERC has limitations similar to those harbored by TGC, such as intravenous formulation and disturbance of gastrointestinal (GI) function. Of the adults included in a pharmacokinetic study of ERC, 62.5% (15/24) reported the occurrence of nausea and vomiting (33). These disadvantages discourage the administration of both TGC and ERC in the clinical setting.

OMC, which can be administered both orally and intravenously, presented *in vitro* activity against M. abscessus complex comparable to that of TGC but less strong activity against M. fortuitum. Consistent with a previous study (35), OMC decay was obvious, decreasing to 16.7 to 27.1% of the initial concentration within 5 days in cation-adjusted Mueller-Hinton broth (CAMHB) medium at 37°C (see the supplemental material and Fig. S1), which may be why OMC owned the least decreased value of Log_10_ CFU against M. abscessus subsp. *abscessus* after 4 days’ incubation at 37°C (Fig. 3). A pharmacokinetic study performed by Yang et al. demonstrated that the *C*_max_ of OMC, following administration at 300 mg p.o. once daily, was 1.33 μg/mL (36). For Streptococcus pneumoniae, the recommended dosing could obtain a probability of target attainment (PTA) of >90% in strains with MICs of ≤0.5 μg/mL. Therefore, we presume that OMC could be efficacious in RGM infection treatment, as the majority of the clinical isolates of M. abscessus complex and of the M. fortuitum isolates had MICs of ≤0.5 μg/mL. In addition, OMC showed a good safety profile in the Chinese population, with 12.0% (6/50) reporting intolerance due to GI disturbance (36). Recently, a preliminary study showed that the success rate of the oral OMC-containing regimen in the treatment of M. abscessus infection was 75.0% (9/12) (37). Importantly, OMC showed high safety and good tolerance in the median (interquartile range) duration of 6.2 (4.2 to 11.0) months, as only one patient experienced GI intolerance, which improved with a reduction of the dose from 300 mg daily to 150 mg (37). Due to the dramatically reduced side effects, we deduce that OMC is promising for the treatment of RGM infections. Unfortunately, although the best safety parameters were obtained for SAC in this study and in other reports, SAC demonstrated only mild inhibitory activity against the RGM strains (38, 39). A clinical phase II trial including 285 subjects showed that once-daily oral treatment with SAC was safe and well tolerated at a dosage of 3.0 mg/kg of body weight for 12 weeks. Most importantly, the gastrointestinal adverse effect rate in the SAC group was similar to that in the placebo group (4.1% in the placebo group and 3.3% in the pooled SAC group) (25). Similarly, low adverse event rates were obtained in another phase III clinical trial, i.e., the rates of nausea or vomiting and diarrhea were 7.69% (37/481) and 3.70% (19/513) (24). In the cytotoxicity assay, SAC was the only antibiotic that did not obviously affect the THP-1 cell viability at a concentration of 20 μg/mL. The reported *C*_max_ values of SAC at the dosage of 100 mg/day were between 1.65 μg/mL and 2.09 μg/mL (40), which might still support its use for strains with low MICs. Hence, the outstanding characteristic of good tolerance with high safety for long-term use makes it worthy for treating susceptible RGM cases.

We compared the activities of the four tetracycline antibiotics between M. abscessus subsp. *abscessus* and subsp. *massiliense.* Since we did not have any M. abscessus subsp. *bolletii* in stock, this subspecies was not included.

This study has some limitations. First, all of the clinical strains were collected in a single center and the strain number was small, and thus, the efficacies of the tested tetracyclines against RGM species need further validation on a larger scale. Second, since well-recognized susceptibility testing methods for TGC, ERC, OMC, and SAC have not yet been established, we did not analyze the drug resistance rate of each tested antibiotic. Third, *in vitro* antimicrobial activity does not always reflect the *in vivo* drug response, and therefore, clinical trials are warranted to prove our findings in this study (41).

In conclusion, this study demonstrated that TGC, ERC, and OMC all had very potent *in vitro* antimicrobial activities against M. abscessus complex and M. fortuitum, with OMC being more promising for clinical usage, as supported by the pharmacokinetic/pharmacodynamic (PK/PD) data, safety, and patient compliance. These data provide important insights into the possible clinical applications of tetracycline-class antibiotics in the treatment of RGM infections.

## MATERIALS AND METHODS

### Ethics statement.

All the isolates in our study were obtained from in the Bio-bank in Beijing Chest Hospital, with approval by the Ethics Committee of the Beijing Chest Hospital, Capital Medical University (2021-32-01).

### Reference strains and clinical isolates.

The mycobacterial reference strains, including 27 RGM species, were obtained either from the American Type Culture Collection (ATCC) or from the German Collection of Microorganisms (DSM). The species constitution of these reference strains is listed in Table 1. Clinical mycobacterial isolates of certain RGM species, stored in the Bio-bank in Beijing Chest Hospital (Beijing, China), were recruited. All the mycobacterial isolates included were preliminarily categorized as NTM with growth using *p*-nitrobenzoic acid-containing Löwenstein-Jensen (LJ) medium at a concentration of 500 μg/mL. The strains were then identified at species level by sequencing of 16S rRNA, *hsp65*, *rpoB*, and 16S–23S rRNA internal transcribed spacer genes. In total, 121 clinical RGM isolates were recruited, including M. abscessus subsp. *abscessus* (*n* = 44) and M. abscessus subsp. *massiliense* (*n = *29) isolates, as well as 43 M. fortuitum isolates and 5 M. chelonae isolates. All of the included isolates were stored at −80°C and subcultured on LJ medium before performing antibiotic susceptibility testing.

### MIC testing.

TGC, ERC, OMC, and SAC were purchased from MedChemExpress, USA, and were dissolved in dimethyl sulfoxide (DMSO) with a concentration of 25.6 mg/mL for preparing the stock solution. MICs were determined by the broth microdilution method according to the guidelines of the Clinical and Laboratory Standards Institute (CLSI) (42). Cation-adjusted Mueller-Hinton broth (CAMHB) was used for testing the MICs of RGM isolates. The inoculum was prepared with fresh culture grown on LJ medium. The antimicrobial concentrations tested ranged from 0.0078 μg/mL to 8 μg/mL. Briefly, stock solutions of the compounds were 1,600-fold diluted with CAMHB at its highest concentration and then serially 2-fold diluted in clear 96-well microtiter plates. The bacterial culture growing on the LJ medium was homogenized with 5% Tween 80 and adjusted to McFarland 0.5. Then, the cultures were diluted 200-fold and inoculated with an aliquot of 100 μL per well. The strains were incubated at 37°C for 3 days, except for Mycobacterium peregrinum strain DSM43271, which was incubated at 30°C, and then 30 μL resazurin (0.02%, wt/vol) was added to each well and the plates were reincubated for an additional 24 h at 37°C. Finally, the color change was read visually. The MIC was defined as the lowest concentration of compound that prevented the color change from blue to pink (43).

### MBC testing.

The minimum bactericidal concentrations (MBCs) of TGC, ERC, OMC, and SAC were determined by CFU enumeration on MH agar plates against M. abscessus subsp. *abscessus* strain ATCC 19977, M. fortuitum strain ATCC 6841, five M. abscessus subsp. *abscessus* clinical isolates and four subsp. *massiliense* clinical isolates (including six with rough morphology and three with smooth morphology, as there are identified differences in susceptibility to certain antibiotics between smooth and rough variants [44]; however, there were no statistically significant differences in the decreased value of Log_10_ CFU for tetracyclines against M. abscessus subsp. *abscessus* and subsp. *massiliense* in our study), and four M. fortuitum clinical isolates (including two isolates with SAC MICs of 1 μg/mL). In this study, the antimicrobial concentrations included for MBC determination ranged from 1× MIC to 16× MIC. After incubation with the antibiotics for 3 days, 100 μL of bacterial culture from each well was 10-fold serially diluted, inoculated onto Mueller-Hinton (MH) agar plates, and incubated at 37°C. Determination of CFU/mL was performed 4 to 6 days later. The MBC value was defined as the lowest concentration of the drug that reduced the initial bacterial load by ~99.9% (3 log) CFU (45). An antibiotic was regarded as bactericidal when the MBC/MIC ratio was 4 or less; otherwise, it was considered a bacteriostatic agent (44).

### Cytotoxicity assay.

The cytotoxicities of TGC, ERC, OMC, and SAC in the THP-1 macrophage cell line were determined by cell counting kit-8 (CCK-8) assay (Solarbio life sciences, China). The THP-1 cells were purchased from Wuhan Procell Life Science & Technology Co., Ltd., and cultured in RPMI 1640 supplemented with 10% heat-inactivated fetal bovine serum (FBS) (lot no. 2393124RP; Gibco, United States) at 37°C and 5% CO_2_. Cells were plated into a 96-well plate at a density of 3* × *10^4^ cells per well and incubated with 200 ng/mL phorbol myristate acetate (PMA). The macrophages were differentiated over 48 h, the PMA removed, and the cells washed twice and finally treated with the four tetracyclines at different concentrations (5 mg/L, 10 mg/L, 20 mg/L, and 40 mg/L) in RPMI 1640 supplemented with 5% FBS. After incubation for another 24 h, 100 μL of medium containing 10% CCK-8 solution was added to each well and the culture maintained for an additional 2 h. Finally, a Multiskan FC microplate reader (Thermo Fisher, USA) was used to measure the absorbance at 450 nm. According to the kit’s instructions, cell viability in drug-treated groups was defined as the percentage of the value for control group cells after eliminating the impact of background absorbance.

### Quality control.

Quality control for the novel tetracyclines and TGC was performed using M. peregrinum strain DSM43271 and the quality control ranges recommended by the CLSI for the tetracyclines (46). Acceptable MIC ranges for M. peregrinum DSM43271 are 0.03 μg/mL to 0.25 μg/mL for TGC and 0.015 μg/mL to 0.3 μg/mL for ERC (47). All quality control results were within the acceptable ranges.

### Statistical analysis.

Analysis was performed using the statistical software SPSS version 22.0 (SPSS, Inc., Chicago, IL, USA). All tests of significance were two tailed, and a *P* value of <0.05 was considered statistically significant. The IC_50_, maximal inhibitory effect (*E*_max_), and EC_50_ values of tetracyclines were analyzed using Prism version 9 (GraphPad Software) with tests as described in the figure legends. Sample sizes and *P* values are cited in the figures and figure legends.
