# Supplementary material for: In Vitro Antimicrobial Activities of Tigecycline, Eravacycline, Omadacycline, and Sarecycline against Rapidly Growing Mycobacteria
Source: Microbiol Spectr. 2022 Dec 8;11(1):e03238-22. doi: 10.1128/spectrum.03238-22 (PMC9927410; doi:10.1128/spectrum.03238-22)
Supplement: Supplemental file 1 — Supplemental material. Download spectrum.03238-22-s0001.pdf, PDF file, 0.7 MB [file spectrum.03238-22-s0001.pdf]

Supplementary data

Supplementary Online Methods.

Supplementary Results.

Figure S1. Decline in Omadacycline concentration with time in CAMHB at 37°C

SUPPLEMENTARY ONLINE METHODS

Omadacycline stability monitored in CAMHB by high-pressure liquid chromatography/tandem mass spectrometry (HPLC-MS/MS)

Standard solutions. The stock solution of OMC and zaleplon, used as internal standard (IS), were dissolved at 1 mg/mL into Dimethyl sulfoxide (DMSO) (Sigma, Germany). The stock solution was diluted to prepare a nine-point calibration curve as follows. Briefly, the stock solution was diluted to a series of concentrations (19.53125, 39.0625, 78.125, 156.25, 312.5, 625, 1250, 2500, 5000and 10000 µg/L) with OMC-free CAMHB. Next, 10µl IS (2000 µg/L) was added into 100µl different OMC concentrations solutions. And then, a 4-fold volume of methanol was fortified into these mixtures for deproteinization. After centrifugation at 3500×g at 4°C for 15 min, the supernatant was separated for further analysis.

Sample preparation. Stability was monitored of four levels (0.25, 0.5, 1, and 2 mg/L) with three replicates of each group. Samples were prepared by adding the appropriate volume of stock solution into the OMC-free CAMH at 37°C. And 10µl IS (2000 µg/L) was added into 100µl sample supernatant before deproteinization. All samples were collected and analyzed on day 1, 3, 5 and 7.

Instrumentation and chromatographic conditions. Chromatography was performed on the HPLC system (Agilent Technologies, the United State) that including an autosampler (G1329A), a triple quadrupole mass spectrometer (G6420A) and a column heater (G1316A). Electrospray ionization in the positive mode (ESI+) was used for all samples. Chromatographic separation was performed with an Agilent ZORBAX SB-C18 column (2.1 x 50 mm; Agilent Technologies, the United State) for OMC and the column chamber was held at 45 °C. The mobile phases A 0.1% Formic Acid in H_2O and B 0.1% FA in MeOH, were delivered at a flow rate of 0.6 mL/min (MPA:MPB 65:35, v/v) [ref]. The multiple reaction monitoring (MRM) transition was 557.5→ 453.3 ; F: 130V, CE: 26V for OMC, 306.1→ 236.1; F: 150V, CE: 29V for IS. Finally, 5µl liquid was applied on HPLC-MS/MS.

Result

The OMC declined to 52.2-70.3% of initial concentrations after 24h incubation at 37 °C, which was ranged 32.7-53.2% after 72h. Morever, these values were decreased remarkably to 16.7-27.1% (except 0.25 mg/L group) on day 5, while the concentrations were stable between day 5 to 7. Calibration curves in the range of 19.53125–10000 µg/L for OMC were established (r2 = 0.9992).

[ref] Chapagain M, Pasipanodya JG, Athale S et al. Omadacycline efficacy in the hollow fibre system model of pulmonary *Mycobacterium avium* complex and potency at clinically attainable doses. J Antimicrob Chemother. 2022 May 29;77(6):1694-1705. doi: 10.1093/jac/dkac068.

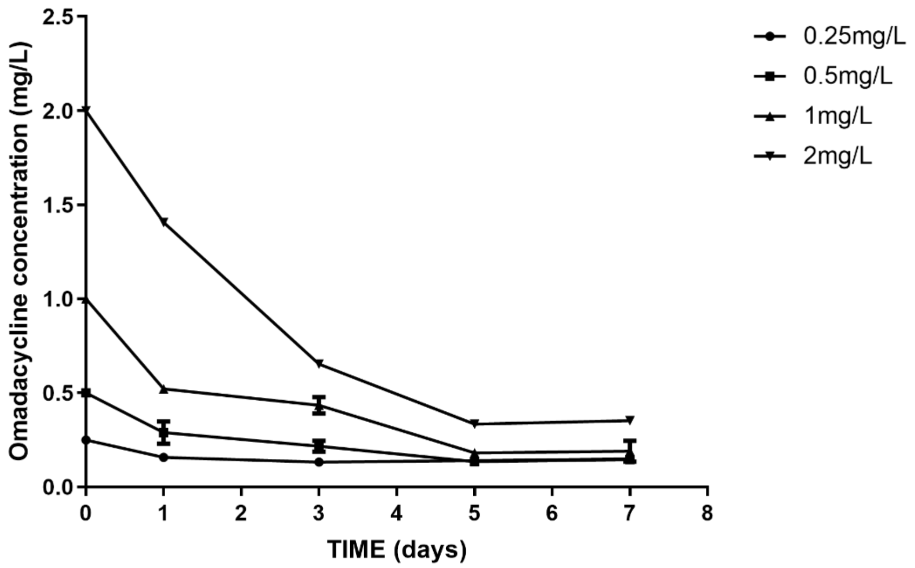

Figure S1. Decline in omadacycline concentration with time in CAMHB at 37℃. All data are shown as the means±SD(n=3).

Supplementary Table S1: MICs of Tigecycline, Eravacycline, Omadacycline, and Sarecycline against the 44 *M. abscessus* subsp. *abscessus* isolates, 29 *M. abscessus* subsp. *massiliense* isolates and 43 *M. fortuitum* isolates.

No.Isolate	Subspecies	MIC (μg/ml)			
		Tigecycline	Eravacycline	Omadacycline	Sarecycline
10	*M. abscessus* subsp. *abscessus*	0.125	0.0625	0.5	>8
39	*M. abscessus* subsp. *abscessus*	0.25	0.0625	0.25	>8
83	*M. abscessus* subsp. *abscessus*	0.125	0.03125	0.25	>8
104	*M. abscessus* subsp. *abscessus*	0.5	0.125	0.5	>8
111	*M. abscessus* subsp. *abscessus*	0.5	0.0625	0.5	1
113	*M. abscessus* subsp. *abscessus*	0.25	0.0625	0.25	>8
115	*M. abscessus* subsp. *abscessus*	0.25	0.125	0.5	>8
121	*M. abscessus* subsp. *abscessus*	0.25	0.0625	0.5	>8
123	*M. abscessus* subsp. *abscessus*	0.0625	0.03125	0.125	0.5
124	*M. abscessus* subsp. *abscessus*	0.25	0.0625	0.25	>8
128	*M. abscessus* subsp. *abscessus*	0.25	0.03125	0.25	>8
130	*M. abscessus* subsp. *abscessus*	0.0625	0.015625	0.125	4
135	*M. abscessus* subsp. *abscessus*	0.5	0.0625	0.5	>8
146	*M. abscessus* subsp. *abscessus*	0.0625	0.015625	0.125	0.125
164	*M. abscessus* subsp. *abscessus*	0.125	0.03125	0.25	>8
168	*M. abscessus* subsp. *abscessus*	0.25	0.0625	0.25	>8
170	*M. abscessus* subsp. *abscessus*	0.25	0.03125	0.5	>8
172	*M. abscessus* subsp. *abscessus*	0.25	0.0625	0.5	>8
263	*M. abscessus* subsp. *abscessus*	0.25	0.125	0.5	>8
269	*M. abscessus* subsp. *abscessus*	0.25	0.0625	0.5	>8
272	*M. abscessus* subsp. *abscessus*	0.125	0.015625	0.125	>8
273	*M. abscessus* subsp. *abscessus*	0.125	0.03125	0.25	>8
276	*M. abscessus* subsp. *abscessus*	0.5	0.0625	0.5	>8
277	*M. abscessus* subsp. *abscessus*	0.25	0.03125	0.25	>8
280	*M. abscessus* subsp. *abscessus*	0.5	0.0625	0.5	>8
657	*M. abscessus* subsp. *abscessus*	0.25	0.015625	0.25	8
665	*M. abscessus* subsp. *abscessus*	0.0625	0.03125	0.25	>8
666	*M. abscessus* subsp. *abscessus*	0.5	0.0625	0.25	8
682	*M. abscessus* subsp. *abscessus*	0.0625	0.015625	0.0625	0.125
690	*M. abscessus* subsp. *abscessus*	0.5	0.125	1	>8
696	*M. abscessus* subsp. *abscessus*	0.0625	0.015625	0.125	2
702	*M. abscessus* subsp. *abscessus*	0.0625	0.015625	0.125	2
711	*M. abscessus* subsp. *abscessus*	0.0625	0.015625	0.0625	2
715	*M. abscessus* subsp. *abscessus*	0.125	0.0625	0.25	4
736	*M. abscessus* subsp. *abscessus*	0.25	0.0625	0.5	>8
747	*M. abscessus* subsp. *abscessus*	0.5	0.25	1	>8
778	*M. abscessus* subsp. *abscessus*	0.25	0.0625	0.25	1
779	*M. abscessus* subsp. *abscessus*	0.125	0.0625	0.25	4
800	*M. abscessus* subsp. *abscessus*	0.5	0.0625	1	4
801	*M. abscessus* subsp. *abscessus*	0.125	0.03125	0.25	2
811	*M. abscessus* subsp. *abscessus*	0.125	0.03125	0.5	>8
847	*M. abscessus* subsp. *abscessus*	0.25	0.0625	0.5	>8
848	*M. abscessus* subsp. *abscessus*	1	0.125	0.5	>8
859	*M. abscessus* subsp. *abscessus*	0.0625	0.03125	0.125	8
17	*M. abscessus* subsp. *massiliense*	0.125	0.0625	0.5	8
26	*M. abscessus* subsp. *massiliense*	0.25	0.03125	0.25	>8
105	*M. abscessus* subsp. *massiliense*	0.5	0.0625	0.5	>8
106	*M. abscessus* subsp. *massiliense*	0.25	0.0625	1	>8

107	*M. abscessus* subsp. *massiliense*	1	0.125	0.5	>8
108	*M. abscessus* subsp. *massiliense*	0.25	0.0625	0.5	>8
109	*M. abscessus* subsp. *massiliense*	1	0.0625	0.5	>8
112	*M. abscessus* subsp. *massiliense*	0.125	0.015625	0.125	2
116	*M. abscessus* subsp. *massiliense*	0.25	0.015625	0.125	8
119	*M. abscessus* subsp. *massiliense*	0.5	0.0625	1	>8
120	*M. abscessus* subsp. *massiliense*	0.5	0.0625	1	>8
122	*M. abscessus* subsp. *massiliense*	0.125	0.015625	0.25	>8
126	*M. abscessus* subsp. *massiliense*	0.0625	0.015625	0.125	8
131	*M. abscessus* subsp. *massiliense*	0.5	0.0625	0.5	>8
133	*M. abscessus* subsp. *massiliense*	0.5	0.0625	0.5	8
234	*M. abscessus* subsp. *massiliense*	0.25	0.125	0.5	>8
242	*M. abscessus* subsp. *massiliense*	1	0.25	1	>8
274	*M. abscessus* subsp. *massiliense*	0.25	0.0625	0.25	>8
659	*M. abscessus* subsp. *massiliense*	0.25	0.0625	0.25	>8
674	*M. abscessus* subsp. *massiliense*	0.125	0.015625	0.25	>8
678	*M. abscessus* subsp. *massiliense*	0.25	0.0625	0.25	8
679	*M. abscessus* subsp. *massiliense*	0.25	0.0625	0.5	>8
704	*M. abscessus* subsp. *massiliense*	0.03125	0.015625	0.25	>8
740	*M. abscessus* subsp. *massiliense*	0.125	0.03125	0.25	>8
749	*M. abscessus* subsp. *massiliense*	0.25	0.125	0.5	8
759	*M. abscessus* subsp. *massiliense*	0.25	0.0625	0.5	4
776	*M. abscessus* subsp. *massiliense*	0.25	0.0625	0.25	8
783	*M. abscessus* subsp. *massiliense*	0.25	0.0625	0.5	>8
802	*M. abscessus* subsp. *massiliense*	0.25	0.0625	0.25	2
1	*M. fortuitum*	0.03125	0.015625	0.25	>8
4	*M. fortuitum*	0.0625	0.015625	0.5	>8
5	*M. fortuitum*	0.015625	0.015625	0.5	4
6	*M. fortuitum*	0.015625	0.007813	0.5	4
7	*M. fortuitum*	0.03125	0.015625	0.25	8
9	*M. fortuitum*	0.0625	0.015625	0.5	>8
31	*M. fortuitum*	0.015625	0.007813	0.125	>8
101	*M. fortuitum*	2	0.25	8	4
103	*M. fortuitum*	0.015625	0.007813	0.25	>8
114	*M. fortuitum*	0.015625	0.007813	0.25	>8
117	*M. fortuitum*	0.03125	0.015625	0.5	1
118	*M. fortuitum*	0.015625	0.007813	0.25	>8
129	*M. fortuitum*	0.03125	0.007813	0.25	>8
267	*M. fortuitum*	0.015625	0.007813	0.25	>8
433	*M. fortuitum*	0.0625	0.015625	0.5	>8
446	*M. fortuitum*	0.015625	0.007813	0.25	>8
534	*M. fortuitum*	0.015625	0.007813	0.125	>8
658	*M. fortuitum*	0.03125	0.007813	0.5	>8
925	*M. fortuitum*	0.015625	0.007813	0.125	1
987	*M. fortuitum*	0.015625	0.015625	0.5	>8
1021	*M. fortuitum*	0.25	0.03125	0.25	>8
1265	*M. fortuitum*	0.25	0.0625	0.5	>8
1280	*M. fortuitum*	0.25	0.0625	0.5	>8
1316	*M. fortuitum*	0.0625	0.015625	0.5	>8
1594	*M. fortuitum*	0.0625	0.007813	0.5	>8
1989	*M. fortuitum*	0.0625	0.015625	0.25	0.5
1990	*M. fortuitum*	0.03125	0.015625	0.25	0.5
1991	*M. fortuitum*	0.03125	0.015625	0.25	0.5

2088	*M. fortuitum*	0.0625	0.015625	0.5	>8
2148	*M. fortuitum*	0.03125	0.007813	0.25	0.5
2237	*M. fortuitum*	0.0625	0.015625	0.5	>8
2297	*M. fortuitum*	0.0625	0.015625	0.5	2
2480	*M. fortuitum*	0.0625	0.015625	0.5	1
2481	*M. fortuitum*	0.03125	0.015625	0.25	>8
2482	*M. fortuitum*	0.0625	0.015625	0.5	>8
2483	*M. fortuitum*	0.03125	0.015625	0.5	>8
2484	*M. fortuitum*	0.03125	0.007813	0.25	1
2624	*M. fortuitum*	0.03125	0.007813	0.5	>8
2625	*M. fortuitum*	0.0625	0.015625	0.5	>8
2696	*M. fortuitum*	0.125	0.03125	0.5	>8
2697	*M. fortuitum*	0.03125	0.007813	0.125	0.25
2698	*M. fortuitum*	0.0625	0.015625	0.5	>8
2699	*M. fortuitum*	0.0625	0.015625	0.5	>8

Table S2. Minimum inhibitory concentration (MIC), minimum bactericidal concentration (MBC) values, and antibacterial activities of TGC, ERC, OMC and SRC against *M. abscessus* subsp. *abscessus* and subsp. *massiliense.*

Isolate	Morphotype	Tigecycline MIC (μg/ml)			Eravacycline (μg/ml)			Omadacycline (μg/ml)			Sarecycline (μg/ml)		
		MIC	MBC	MIC/MBC ratio	MIC	MBC	MIC/MBC ratio	MIC	MBC	MIC/MBC ratio	MIC	MBC	MIC/MBC ratio
ATCC 19977[a]	Smooth	0.25	4	16	0.0625	>1	>16	0.25	>4	>16	>8	—	—
M. abscessus subsp. *massiliense* 119	Smooth	0.5	>8[b]	>16	0.0625	>1	>16	1	>16	>16	>8	—	—
M. abscessus subsp. *abscessus* 121	Smooth	0.25	4	16	0.0625	>1	>16	0.5	>8	>16	>8	—	—
M. abscessus subsp. *massiliense* 106	Rough	0.25	>4	>16	0.0625	>1	>16	1	>16	>16	>8	—	—
M. abscessus subsp. *massiliense* 108	Rough	0.25	>4	>16	0.0625	>1	>16	0.5	>8	>16	>8	—	—
M. abscessus subsp. *massiliense* 120	Rough	0.5	8	16	0.0625	>1	>16	1	>16	>16	>8	—	—
M. abscessus subsp. *abscessus* 123	Smooth	0.0625	—[c]	—	0.0313	—	—	0.125	—	—	0.5	>8	>16
M. abscessus subsp. *abscessus* 146	Smooth	0.0625	—	—	0.0156	—	—	0.125	—	—	0.125	2	16
M. abscessus subsp. *abscessus* 682	Smooth	0.0625	—	—	0.0156	—	—	0.0625	—	—	0.125	2	16
M. abscessus subsp. *abscessus* 778	Rough	0.25	—	—	0.0625	—	—	0.25	—	—	1	>16	>16

[a]ATCC 19977 is the reference stain of *M. abscessus* subsp. *abscessus*.

[b]MIC values greater than the 8 mg/L value that was tested.

[c]not done.

Table S3. Minimum inhibitory concentration (MIC), minimum bactericidal concentration (MBC) values, and antibacterial activities of TGC, ERC, OMC and SRC against *M. fortuitum*.

Isolate	Tigecycline MIC (μg/ml)			Eravacycline (μg/ml)			Omadacycline (μg/ml)			Sarecycline (μg/ml)		
	MIC	MBC	MIC/MBC ratio	MIC	MBC	MIC/MBC ratio	MIC	MBC	MIC/MBC ratio	MIC	MBC	MIC/MBC ratio
ATCC 6841[a]	0.015	>0.25	>16	0.0078	>0.125	>16	0.5	4	8	2	>32	>16
1	0.03	0.5	16	0.015	>0.25	>16	0.25	2	8	>8[b]	—[c]	—
1021	0.25	>4	>16	0.03	>0.5	>16	0.25	4	16	>8	—	—
117	0.03	0.25	8	0.015	>0.25	>16	0.5	4	8	1	16	16
925	0.015	>0.25	>16	0.0078	>0.125	>16	0.125	1	8	1	>16	>16

[a]ATCC 6841 is the reference stain of *M. fortuitum*.

[b]MIC values greater than the 8 mg/L value that was tested.

[c]not done.
